# Weak phonon scattering effect of twin boundaries on thermal transmission

**DOI:** 10.1038/srep19575

**Published:** 2016-01-29

**Authors:** Huicong Dong, Jianwei Xiao, Roderick Melnik, Bin Wen

**Affiliations:** 1 State Key Laboratory of Metastable Materials Science and Technology, Yanshan University, Qinhuangdao 066004, China; 2 The MS2Discovery Interdisciplinary Research Institute, Wilfrid Laurier University, 75 University Ave. West, Waterloo, Ontario, Canada N2L 3C5

## Abstract

To study the effect of twin boundaries on thermal transmission, thermal conductivities of twinned diamond with different twin thicknesses have been studied by NEMD simulation. Results indicate that twin boundaries show a weak phonon scattering effect on thermal transmission, which is only caused by the additional twin boundaries’ thermal resistance. Moreover, according to phonon kinetic theory, this weak phonon scattering effect of twin boundaries is mainly caused by a slightly reduced average group velocity.

As a critically important material property, thermal conductivity of nanocrystalline materials is of quite general interest due to its significance in many technological applications, such as bioMEMs^1^, gas-turbine-blade coatings^2^, and micro-/nanoelectromechanical devices^2^ etc. Generally, with decreasing grain size of nanocrystalline materials, thermal conductivity is greatly decreased due to drastically reduced phonon mean free path arising from grain boundaries’ strong scattering effect^3^,^4^,^5^,^6^. It is well known that twin boundaries, a special type of grain boundaries^7^, can improve materials’ strength^8^ and hardness^9^,^10^, like conventional grain boundaries^8^. However, unlike the effect of twin boundaries on mechanical properties, the effect of twin boundaries on phonon thermal transmission is unclear and it is still in debate. For example, Mitchell’s InTl alloys’ thermal transmission experiment^11^ indicated that the existence of micrometer-scale twin boundaries can reduce InTl alloys’ thermal conductivity due to additional phonon scattering at twin boundaries. However, this scattering effect was much weaker than that of conventional grain boundaries (only about 20% of conventional grain boundaries’ scattering effect). Besides, Zhan’s reverse non-equilibrium molecular dynamics simulation on thermal conductivities of nanotwinned Si nanowires demonstrated that the twin boundaries had less effect on the thermal conductivity of Si nanowires (reduced about 8% of perfect Si nanowires’ thermal conductivity)^12^. While for Li’s non-equilibrium molecular dynamics (NEMD) simulation results on nanotwinned ferroic films’ thermal conductivity^13^, the existence of twin boundaries perpendicular to heat flow can greatly reduce nanotwinned ferroic films’ thermal conductivity (reduced about 97% of perfect ferroic film’s thermal conductivity). Li contributed this great reduction of thermal conductivity to the strong twin boundaries’ scattering effect on the longitudinal phonon transmission.

Although the phonon scattering effect of twin boundaries on thermal transmission has been verified by the above studies, the degree of this effect shows serious inconsistencies. To clear this issue, in this work, the thermal transmission of twinned diamond with different twin thicknesses (*0.62 nm*~*9.92 nm*) has been investigated by using the NEMD simulation method. Our results demonstrate that twin boundaries show a much weaker phonon scattering effect on thermal transmission than that of conventional grain boundaries.

In this paper, to avoid the effect of model size on thermal conductivity of bulk twinned diamond and bulk perfect diamond, thermal conductivities of these two kinds of structures with different model sizes have been calculated^14^,^15^. Due to the existence of the linear relationship between the inverse of thermal conductivity (*1/K*) and the inverse of model size (*1/L*) (detailed deduction can be found in Supplement discussion 1), bulk crystal thermal conductivities can be deduced by extrapolating this linear relationship, as shown in Fig. 1 (a). By using this extrapolation method, the thermal conductivities of bulk twinned diamond with different twin thicknesses (*D*), as well as that of the bulk perfect diamond can be calculated, and they are plotted in Fig. 1(b). As can be seen in Fig. 1 (b), the thermal conductivity of bulk twinned diamond is smaller than that of the bulk perfect diamond. With increasing twin thickness, thermal conductivity of bulk twinned diamond is increased. When the twin thickness is increased from *3.72 to 9.92 nm*, bulk twinned diamond thermal conductivity *K*_*b−td*_ varies from *1684* to *1846 W/mK*, which is about *87.7*~*96%* of our calculated thermal conductivity for the bulk perfect diamond *K*_*b-per*_(*1920 W/mK*). It is well known that the effect of twin boundaries on mechanical properties can be comparable with that of conventional grain boundaries^8^, similarly, to explore the effect of twin boundaries on thermal transmission, bulk twinned diamond thermal conductivity has been compared with that of nanocrystalline diamond in our previous work^6^. By comparison, as shown in Fig. 1 (b), when the grain size of nanocrystalline diamond ranges from *3.6* to *11.1 nm*, the thermal conductivity of the nanocrystalline diamond *K*_*nano*_ increases from *10.2* to *28.3 W/mK*^6^, and these values are only about *0.5% to1.5%* of bulk perfect diamond thermal conductivity, much smaller than bulk twinned diamond thermal conductivity with similar sizes of twin thickness. Despite significant difference between the distribution of grain boundaries in the twinned and nanocrystalline diamond, since thermal transmission is only affected by grain boundaries perpendicular to heat flux^13^, and anisotropy of structures shows low effect on thermal transmission^6^, this great difference between the bulk twinned and the nanocrystalline diamond thermal conductivity is mainly caused by the different structures of twin boundaries and conventional grain boundaries. Therefore, it can be concluded that although the twin thicknesses in twinned diamond is in the same order of magnitude with the grain sizes of nanocrystalline diamond, the degree of the effects respectively caused by twin boundaries and conventional grain boundaries on heat transmission is substantially different.

Due to tandem properties of thermal transmission^16^, thermal resistance of twinned diamond can be divided into two tandem parts: one is intragranular part, and the other is intergranular part (i. e. twin boundaries), that is


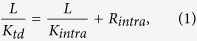


where *L*/*K*_*td*_ is the thermal resistance (*m*^*2*^*K/W*) for twinned diamond with a model size of *L* (*meter*). *L*/*K*_*intra*_ and *R*_*inter*_ are the thermal resistances (*m*^*2*^*K/W*) of intragranular and intergranular parts for this structure, respectively.

In Eq. (1), the intergranular thermal resistance *R*_*inter*_ is the total thermal resistances of all twin boundaries. In our analysis, twin boundary thermal resistances are considered as independent ones which are placed in series. Thus, *R*_*inter*_ can be described as


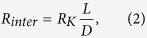


where *D* is the twin thickness, and *R*_*K*_ is the thermal resistance (*m*^*2*^*K/W*) for one twin boundary.

By combining Eq.(1) and Eq.(2), we can obtain


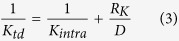


Because of the coincident radial distribution functions of twinned diamond and perfect diamond (as shown in supplementary Figure S1), it can be deduced that the C-C bond lengths of the twinned and perfect diamond are similar, and further the phonon-phonon scattering process in the intragranular part of twinned diamond is the same as that in the perfect diamond. Therefore, the intragranular thermal conductivity *K*_*intra*_ for twinned diamond can be considered as perfect diamond thermal conductivity. Since 1/*K*_*td*_ and 1/*K*_*per*_ are both parameters linearly related to *1/L*, Eq. (3) leads to


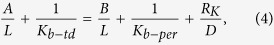


where *A* is the slope of the inverse of twinned diamond thermal conductivity (*1/K*_*td*_) for the inverse of model size (*1/L*), and *B* is the slope of the inverse of perfect diamond thermal conductivity (*1/K*_*per*_) for the inverse of model size (*1/L*). When *L* is infinite in Eq. (4), thermal conductivity of bulk twinned diamond (*K*_*b−td*_) can be calculated by the following relationship


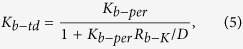


where *R*_*b−K*_ is the twin boundary thermal resistance in bulk twinned diamond.

Moreover, a linear relationship in twinned diamond between *R*_*K*_*/D* and *1/L* can be easily deduced as


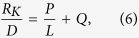


where *P* is the slope of intergranular thermal resistance (*R*_*K*_*/D*) for the inverse of model size (*1/L*), and *Q* is the intercept of this regression line which is used to describe the linear relationship between *R*_*K*_*/D* and *1/L*. Thus, for a twinned diamond with a certain twin thickness *D*, it also keeps a linear relationship between *R*_*K*_ and *1/L*.

The twin boundary thermal resistance *R*_*K*_ can be calculated by the following equation^17^


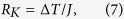


where Δ*T* is the temperature drop across the twin boundary, and *J* is the introduced heat flux.

To calculate twin boundary thermal resistance *R*_*K*_, simulations of Δ*T* have been conducted on twinned diamond with different model sizes (*L*), and results for Δ*T* in twinned diamond with *D = 9.9 nm* has been shown in Figure S2. It can be seen that with increasing model size, Δ*T* are decreased. Same trends can be found in twinned diamond with other twin thicknesses (*D*) as well (such as *3.72 nm* and *7.44 nm*). By using Eq. (7), calculated results for *R*_*K*_at different model sizes (*L*) are plotted in Fig. 2. The predicted linear relationship between twin boundary thermal resistance *R*_*K*_and inverse of model size *1/L* can also be seen from Fig. 2.

The twin boundary thermal resistance in bulk twinned diamond (*R*_*b-K*_) can be obtained by extrapolating *1/L* to 0, and in the range of our computation accuracy, *R*_*b-K*_ remains a constant despite different twin thicknesses, as shown in Fig. 2. Its value is about 2 × 10^−13^ *m*^2^ *K*/*W*, three orders of magnitude lower than the conventional grain boundary thermal resistance in nanocrystalline diamond (1.43 × 10^−10^ *m*^2^ *K*/*W*)^6^, thus the effect of twin boundaries on thermal transmission is much weaker than that of the conventional grain boundaries, and this phenomenon has already been verified by Aubry *et al.* about the grain boundary Kaptiza resistance in silicon^18^.

According to Eq. (3), for bulk twinned diamond with a unit length (*1 meter*), the intragranular thermal resistance *R*_*b-intra*_(or *1/K*_*b-intra*_) can also be calculated by removing the intergranular thermal resistance *R*_*b-inter*_ (or *R*_*b-K*_*/D*) from the bulk twinned diamond thermal resistance *R*_*b-td*_ (or *1/K*_*b-td*_). The plots of calculated intergranular and intragranular thermal resistance for bulk twinned diamond with different twin thickness *D* have been shown in Fig. 3. With increasing twin thickness, the intergranular thermal resistances *R*_*b-inter*_ are decreased due to the decreasing number of twin boundaries, while the intragranular thermal resistances *R*_*b-intra*_ are almost invariable. Moreover, they are all very close to the thermal resistance of the bulk perfect diamond with a unit length *1/K*_*b-per*_ (5.2 × 10^−4^ *m*^2^ *K*/*W*), which implies that the size effect on intragranular thermal resistances in bulk twinned diamond is practically negligible, and this result further confirms that the intragranular thermal conductivity *K*_*b-intra*_ is equal to the bulk perfect diamond thermal conductivity *K*_*b-per*_.

Moreover, Eq. (5) can also be applied in thermal conductivity calculation for other bulk twinned semiconductors. For one kind of bulk twinned structure materials, its thermal conductivity can be easily obtained from its twin thickness *D*, its corresponding bulk perfect crystal thermal conductivity *K*_*b-per*_, and its twin boundary thermal resistance *R*_*b-K*_ in the bulk twinned structure.

For nanocrystalline diamond, both grain sizes and grain boundaries have played a rather significant role in its thermal transmission. But for bulk twinned diamond, sizes of twin thickness almost have no effect on its thermal transmission. In order to provide some insight into thermal transmission mechanisms, the separate contributions from the intergranular thermal resistance *R*_*b-inter*_and intragranular thermal resistance *R*_*b-intra*_ to the bulk twinned diamond (*1 meter*) thermal resistance *R*_*b-td*_ have also been analyzed. The former contribution can be represented by the ratio between the intergranular thermal resistance to the bulk twinned diamond thermal resistance *R*_*b-inter*_*/R*_*b-td*_, and the latter contribution can be represented by the ratio between the intragranular thermal resistance to the bulk twinned diamond thermal resistance *R*_*b-intra*_*/R*_*b−td*_. Calculated results can be found in the inserted table of Fig. 3. With the increasing twin thickness, the value of *R*_*b-inter*_*/R*_*b-td*_ is decreased, while *R*_*b-intra*_*/R*_*b-td*_ is increased. When the twin thickness is increased to *9.92 nm*, *R*_*b-intra*_*/R*_*b-td*_ is 0.96, and *R*_*b-inter*_*/R*_*b-td*_ is only 0.04, in which case the thermal conductivity is very close to the perfect crystal, and the effect of twin boundaries on thermal transmission can be almost ignored. This critical value (*9.92 nm*) in bulk twinned diamond is much smaller than that in the nanocrystalline diamond^6^ (about *10000 nm*), and this inconformity in these two structures also suggests a much weaker phonon scattering effect of twin boundaries than that of conventional grain boundaries.

Since phonons are the primary heat carriers in semiconductors^19^, the weak phonon scattering effect of twin boundaries on thermal transmission has also been analyzed by phonon kinetic theory^4^, which describes the thermal conductivity as


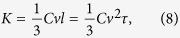


where *C* is the constant volume specific heat capacity, *v* is the average phonon group velocity, *l* is the phonon mean free path, and *τ* is the characteristic relaxation time associated with the phonon scattering process.

For comparison, phonon transmissions in both twinned diamond (*D = 0.62 nm*) and the perfect diamond are analyzed by using phonon kinetic theory. In this work, the heat capacities at *300 K* are calculated (refer to Supplement discussion 2) for these two structures. As shown in Figure S3, D-value between perfect diamond heat capacity and twinned diamond heat capacity is only *−0.02 J/K·mol*, and calculated heat capacities of the two structures are both about *6.3 J/K·mol*. The average phonon group velocity^20^ can be calculated by the following formula:


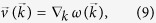


where 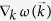 is the gradient between the frequencies and *k* points of both the acoustic and optical branches in the phonon spectra (Fig. 4).The calculated average phonon group velocities of the twinned diamond *v*_*td*_ and the perfect diamond *v*_*per*_ are about *4721 m/s* and *4953 m/s*, respectively. The ratio between 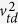 and 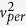 is about 90%. It can be seen that twin boundaries can slightly reduce the average phonon group velocity. Because of a proportional relationship between the relaxation rate (*τ*^−1^) and the square of Grüneisen parameter in the phonon-phonon scattering process, as described by


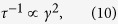


the relaxation time (*τ*) in twinned and perfect diamond can been deduced from the Grüneisen parameter (*γ*)^21^, which, for each mode *i* and frequency *ω*, is defined as


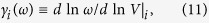


the logarithmic derivative of the phonon angular frequency *ω* with respect to the volume *V* of the crystal. In this paper, the mode- and frequency- averaged Grüneisen parameters (*γ*) for the twinned and perfect diamond have been calculated from the first-principles phonon calculations at three different volumes^22^, and their values are *0.79* and *0.78*, respectively. Further, according to Eq. (10), it can be deduced that the relaxation time of the twinned diamond is about *98%* of that for the perfect diamond. It, therefore, can be concluded that the weak phonon scattering effect of twin boundaries on thermal transmission is caused by both the slightly reduced average phonon group velocity and relaxation time, and the main contribution is arising from the former. According to Lu’s conclusion of lower electrical resistivity of twin boundaries than that of the conventional grain boundaries^8^, our results about weak phonon scattering effect of twin boundaries can be further proven, due to the similar transport properties of phonons and electrons^23^. While in Li’s study about twin boundaries’ effect on thermal transmission of nanotwinned ferroic films, they compared the thermal conductivity of a limited size nanotwinned ferrioc film with that of the perfect ferrioc film, a great reduction of thermal conductivity was thus obtained. They contributed this reduction to the strong phonon scattering of twin boundaries. In fact, in their work, this significant effect of twin boundaries on thermal transmission was caused by the coupling action of twin boundaries scattering and boundary scattering.

In summary, NEMD simulations have been performed to calculate the thermal conductivity of bulk twinned diamond with different twin thicknesses (*0.62*~*9.92 nm*) in order to study the effect of twin boundaries on thermal transmission. Our calculated results indicate that although the bulk twinned diamond thermal conductivity is reduced due to the existence of twin boundaries, twin boundaries show a weak phonon scattering effect on thermal transmission. With the increase of twin thickness, the bulk twinned diamond thermal conductivity is increased. The reduction of the thermal conductivity is only caused by the additional intergranular (twin boundaries’) thermal resistance, which is much smaller than that of conventional grain boundaries (three orders of magnitude lower than the conventional grain boundaries’ thermal resistance). The intragranular thermal resistance in bulk twinned diamond is the same as that of the bulk perfect diamond. According to phonon kinetic theory, this weak phonon scattering effect of twin boundaries is caused by the slightly reduced average phonon group velocity and relaxation time, and the former makes a primary contribution.

## Methods

In order to study the phonon scattering effect of twin boundaries on thermal transmission, a series of cuboids’ [111]-oriented twinned diamond and perfect diamond models have been built. The cross section of these models is *1.75* × *1.25 nm* along 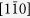 and 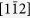 directions, respectively, and the model length along [111] direction ranges from *30* to *150 nm*. The twinned diamond contains a series of parallel 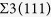
twin boundaries, and Figure S4 (a) is the atomic arrangements of this boundary. A schematic diagram of twinned diamond is shown in Figure S4 (b), and the twin thickness *D* ranges from *0.62* to *9.92 nm*. In this work, thermal conductivity calculation is performed by NEMD simulations^24^,^25^ (refer to Supplement discussion 3) implemented in LAMMPS code^26^, and atomic configurations are visualized by using OVITO package^27^. In our simulated scheme, C-C bonding interactions are described by Tersoff potential^17^, and periodic boundary conditions are imposed in all three directions. The pressure set up is an atmospheric pressure and temperature used is *300 K*. The atomic structures of the twinned and perfect diamond are first optimized in an isothermal-isobaric (NPT) ensemble by using the gradient-based minimization method implemented to minimize the stress for 2×10^5^ steps with a time step of 0.1 fs. After that, a heat flux is imposed on the relaxed structures for 5×10^6^ steps in a micro-canonical (NVE) ensemble to allow the systems’ temperature distribution reaching a steady state. Temperature profiles are obtained by averaging temperatures of simulated atoms in divided slabs^28^ every 1×10^6^ steps. Finally, thermal conductivity can be calculated from the heat flux and temperature gradient by following the Fourier’s heat conduction law^29^.

## Additional Information

**How to cite this article**: Dong, H. *et al.* Weak phonon scattering effect of twin boundaries on thermal transmission. *Sci. Rep.* **6**, 19575; doi: 10.1038/srep19575 (2016).

## Supplementary Material

Supplementary Information

## Figures and Tables

**Figure 1:**
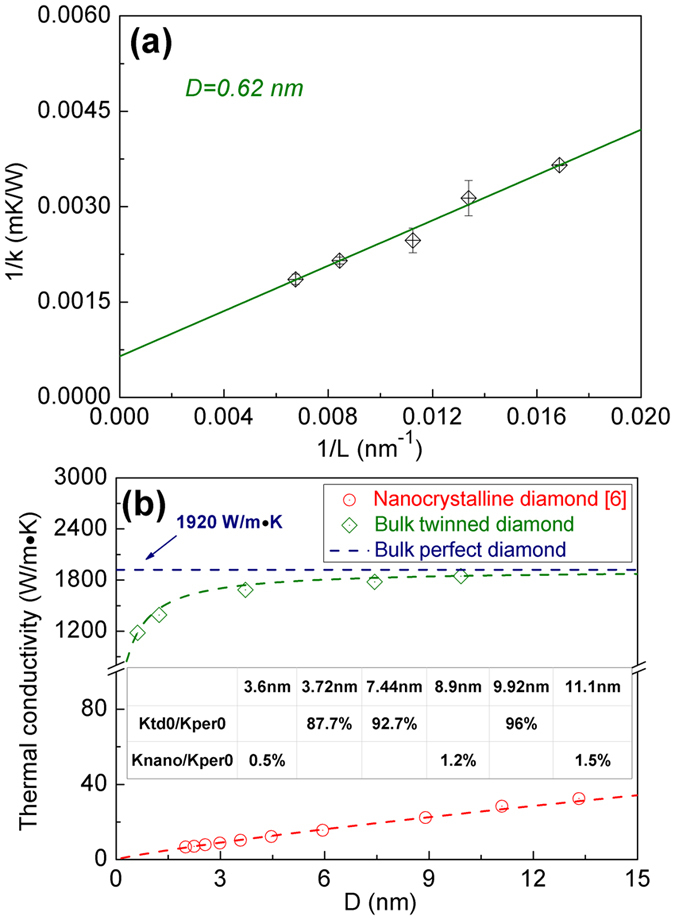
Calculation of bulk twinned diamond thermal conductivity. (**a**) Linear relationship between the inverse of model size (*1/L*) and the inverse of thermal conductivity (*1/K*) for twinned diamond with a twin thickness (*D*) of *0.62 nm*. (**b**) Thermal conductivity of bulk twinned diamond with different twin thicknesses *D* (*0.62 nm ~ 9.92 nm*).

**Figure 2:**
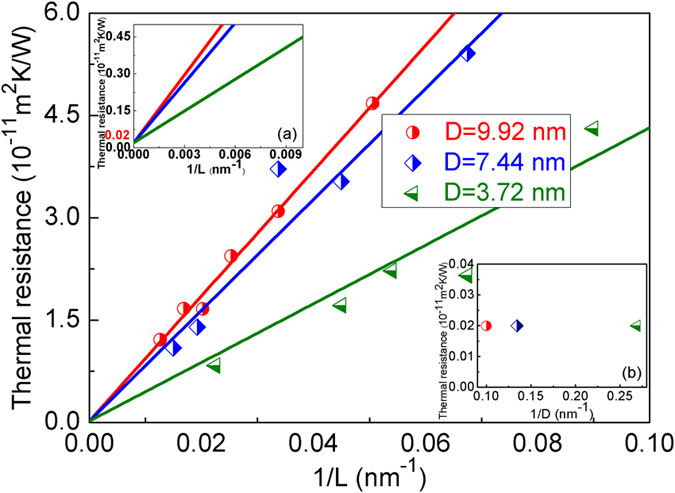
Linear relationship between the inverse of model size (*1/L*) and twin boundary thermal resistance (*R*_*K*_). Insert: (**a**) Magnified linear relationship between the inverse of model size (*1/L*) and twin boundary thermal resistance (*R*_*K*_), when *1/L* tends to *0*, *R*_*K*_*(D = 3.72 nm) = R*_*K*_ *(D = 7.44 nm) = R*_*K*_ *(D = 9.92 nm) = 0.02 *× *10*^*−11*^ *m*^*2*^*K/W*. (**b**) Twin boundary thermal resistance in bulk twinned diamond (*R*_*b–K*_) with different twin thickness (*D*), *R*_*b–K*_ *(D = 3.72 nm) = R*_*b–K*_ *(D = 7.44 nm) = R*_*b–K*_ *(D = 9.92 nm) = 0.02 *× *10*^*−11*^ *m*^*2*^*K/W*.

**Figure 3:**
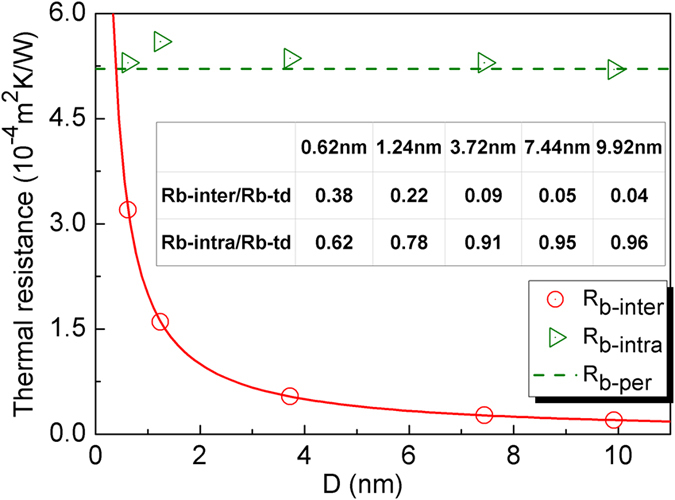
Twin thickness *D* (*0.62 nm ~ 9.92 nm*) dependence of the intergranular (*R*_*b-inter*_) and intragranular thermal resistance (*R*_*b-intra*_) for bulk twinned diamond at *300 K*. Insert table: The separate contribution from the intergranular (*R*_*b-inter*_*/R*_*b−td*_) and intragranular thermal resistances (*R*_*b-inter*_*/R*_*b-td*_) to the total thermal resistance of bulk twinned diamond with different twin thickness *D* (*0.62 nm ~ 9.92 nm*).

**Figure 4:**
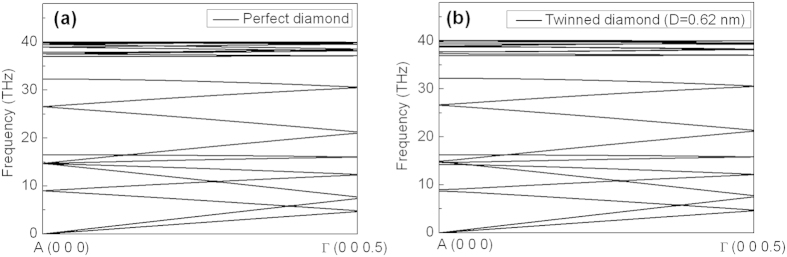
Phonon dispersions of (**a**) perfect diamond and (**b**) twinned diamond (*D = 0.62 nm*) from first-principles calculations along [111].
