# Supplementary material for: Weak phonon scattering effect of twin boundaries on thermal transmission
Source: Sci Rep. 2016 Jan 29;6:19575. doi: 10.1038/srep19575 (PMC4731792; doi:10.1038/srep19575)
Supplement: Supplementary Information [file srep19575-s1.pdf]

Supporting information for

Weak phonon scattering effect of twin boundaries on thermal transmission

Huicong Dong[a], Jianwei Xiao[a], Roderick Melnik[b], Bin Wen[a 1]

[a] State Key Laboratory of Metastable Materials Science and Technology, Yanshan University, Qinhuangdao 066004, China

[b] The MS2Discovery Interdisciplinary Research Institute, Wilfrid Laurier University, 75 University Ave. West, Waterloo, Ontario, Canada N2L 3C5

This file includes:

- **Discussion 1: Bulk crystal thermal conductivity calculation**
- **Discussion 2: Calculation of heat capacity by quasiharmonic approximation (QHA) theory**
- **Discussion 3: Calculation of thermal conductivity by Non-Equilibrium Molecular Dynamics (NEMD) simulation**
- **Figure S1.** Radial distribution functions g(r) for twinned diamond (TD) with different twin thicknesses, perfect diamond (PD) and nanocrystalline diamond (ND) with grain size of *3.6nm*.
- **Figure S2.** Variation of temperature profiles for twinned diamond ($D=9.92$ *nm*) with the variation of model sizes (L). (a) $L=19.84$ *nm*, (b) $L=29.76$ *nm*, (c) $L=39.68$ *nm*, and (d) $L=59.52$ *nm*.
- **Figure S3.** Calculated heat capacity for the twinned ($D=0.62nm$) and perfect diamonds at different temperatures.
- **Figure S4.** (a) Atomic arrangements of a $\sum 3(111)$ twin boundary. (b) Schematic representation for

[a1] Authors to whom any correspondence should be addressed

E-mail address: wenbin@ysu.edu.cn (Bin Wen), Tel: 086-335-8568761

twinned diamond in simulation.

- **Figure S5.** Schematic view of the calculation of thermal conductivity by NEMD method. (a) Diagrammatic sketch of the periodic simulation box as well as the locations of hot and cold regions for NEMD simulation of thermal conduction. (b) Obtained temperature profile along the heat transmission direction.

Discussion 1: Bulk crystal thermal conductivity calculation

According to the kinetic theory of thermal conduction[1], the thermal conductivity of perfect diamond can be expressed as

$$K = \frac{1}{3}C_V v l = \frac{1}{3}C_V v^2 \tau \,, \tag{1}$$

where C_V is the constant volume specific heat capacity for perfect diamond, v is the average phonon group velocity, l is the phonon mean free path, and τ is the characteristic relaxation time associated with the phonon scattering process. In MD simulation, the calculated relaxation time can be decomposed into two parts of contributions[2,3], the "true" bulk contribution, arising from the phonon scattering Umklapp process and point defects[4] in perfect diamond, and a "box" contribution due to the boundaries and the heat source and sink:

$$\tau^{-1} = \tau_{bulk}^{-1} + \tau_{box}^{-1} \,, \tag{2}$$

where τ_{box}^{-1} is expressed as

$$\tau_{box}^{-1} = v / (1/2L) \,, \tag{3}$$

where L is the length of the simulation box. Here the factor of 1/2 arises from the fact that phonon scattering in simulation not only occurs at the heat sink and source regions but also at both boundaries of the simulation box. Therefore, the thermal conductivity of perfect diamond calculated from MD simulations related to the length of the simulation box can be expressed as

$$\frac{1}{K} = \frac{3}{C_V v^2}\tau^{-1} = \frac{3}{C_V v^2}(\tau_{bulk}^{-1} + \frac{2v}{L}) \equiv A + \frac{B}{L} \,. \tag{4}$$

Analysis of the size effect on bulk twinned diamond thermal conductivity calculation, based on this model, is similar to that of the bulk perfect diamond.

Discussion 2: Calculation of heat capacity by quasiharmonic approximation (QHA) theory

In this work, the heat capacities for the twinned and perfect diamonds have been calculated by using the density functional theory combined with quasiharmonic approximation (DFT-QHA) theory[5]. The expression for heat capacity[6] can be expressed as follows:

$$C = \sum_{n,\mathbf{q}} k_B \left(\frac{\hbar\omega_n(\mathbf{q})}{k_B T}\right)^2 \frac{e^{\hbar\omega_n(\mathbf{q})} / k_B T}{(e^{\hbar\omega_n(\mathbf{q})/k_B T} - 1)^2}, \quad (5)$$

where $\hbar$ is the Planck constant, k_B is the Boltzmann constant, T is the temperature, and $\omega_n(\mathbf{q})$ is the phonon frequency of the n-th branch with wave vector **q**.

All the calculations have been performed by implementing the Vienna Ab initio Simulation Package (VASP), which employs the plane-wave basis. The phonon dispersions have been calculated by using the supercell approach with the finite displacement method implemented in the *Phonopy* package[7]. The plane-wave energy cutoff has been set to be *770 eV*, and the electronic energy convergence is 10^{-6} *eV*. In structure relaxations, the force convergence has been set to be 10^{-3}*eV/A*. In phonon calculations, supercells for twinned and perfect diamonds of the hexagonal system both contain *12* atoms, and a Γ-centered $9\times9\times5$ Monkhorst-Pack *k*-point mesh is used to sample the irreducible Brillouin zone.

The calculated results of the heat capacities for the twinned and perfect diamonds at different temperatures are plotted in Figure S3.

Discussion 3: Calculation of thermal conductivity by Non-Equilibrium Molecular Dynamics (NEMD) simulation

Thermal conductivity is calculated using NEMD in this paper. This simulation method is conducted by imposing a heat flux and measuring the induced temperature gradient[8]. As shown in Figure S5 (a), the heat flux is introduced by continuously transferring energy from 'cold' regions, located at the ends of the simulation cell, to 'hot' regions, located in the middle of simulation domain. When the transmission of energy reaches a steady state, thermal conductivity can be calculated from the heat flux and temperature gradient shown in Figure S5 (b) by following the Fourier's heat conduction equation[9]

$$J = -KdT / dx, \tag{6}$$

where K is the thermal conductivity, dT/dx is the temperature gradient averaged over time and space, and J is the heat flux density, which is given by the sum of exchanged energy per unit time and area,

$$J = \frac{1}{2tA} \sum_{transfer} \frac{m}{2} (v_{hot}^2 - v_{cold}^2), \tag{7}$$

where t is the simulation time, A is the cross-sectional area of the simulation system perpendicular to the direction of the heat flux, m is the atom mass, v_{hot} and v_{cold} denote the velocities of the hottest and coldest atoms to be exchanged at each step, and the factor 2 arises from the periodicity.

References

1 Jiang, H., Myshakin, E. M., Jordan, K. D. & Warzinski, R. P. Molecular dynamics simulations of the thermal conductivity of methane hydrate. *J. Phys. Chem. B* **112**, 10207-10216 (2008).

2 Tse, J. S. & White, M. A. Origin of glassy crystalline behavior in the thermal properties of clathrate hydrates: a thermal conductivity study of tetrahydrofuran hydrate. *J. Phys. Chem.* **92**, 5006-5011 (1988).

3 Callaway, J. & von Baeyer, H. C. Effect of point imperfections on lattice thermal conductivity. *Phys. Rev.* **120**, 1149 (1960).

4 Roufosse, M. C. & Klemens, P. Lattice thermal conductivity of minerals at high temperatures. *J. Geophys. Res.* **79**, 703-705 (1974).

5 Otero-de-la-Roza, A. & Luaña, V. Equations of state and thermodynamics of solids using empirical corrections in the quasiharmonic approximation. *Phys. Rev. B* **84**, 184103 (2011).

6 Shao, H. *et al.* First-principles study on the lattice dynamics and thermodynamic properties of Cu2GeSe3. *Europhys. Lett.* **109**, 47004 (2015).

7 Kresse, G. & Hafner, J. Ab initio molecular dynamics for liquid metals. *Phys. Rev. B* **47**, 558 (1993).

8 Bagri, A., Kim, S.-P., Ruoff, R. S. & Shenoy, V. B. Thermal transport across twin grain boundaries in polycrystalline graphene from nonequilibrium molecular dynamics simulations. *Nano Lett.* **11**, 3917-3921 (2011).

9 Ju, S. & Liang, X. Thermal conductivity of nanocrystalline silicon by direct molecular dynamics simulation. *J. Appl. Phys.* **112**, 064305-064305-064307 (2012).

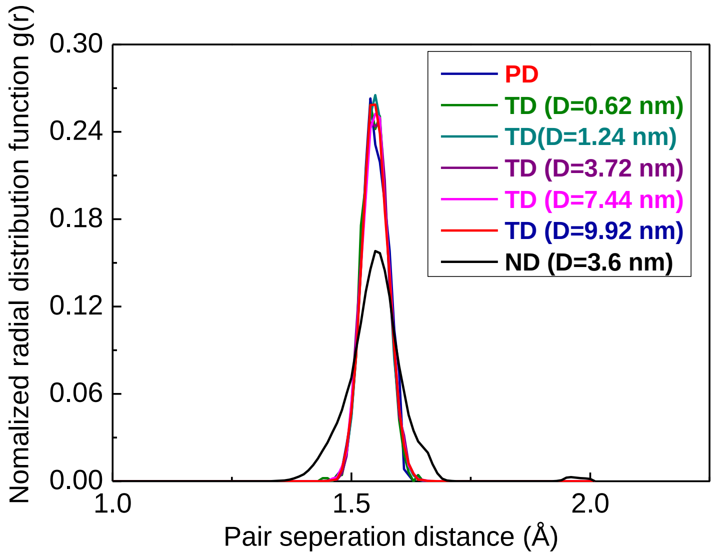

Figure S1. Radial distribution functions g(r) for twinned diamond (TD) with different twin thicknesses, perfect diamond (PD) and nanocrystalline diamond (ND) with a grain size of 3.6 *nm*.

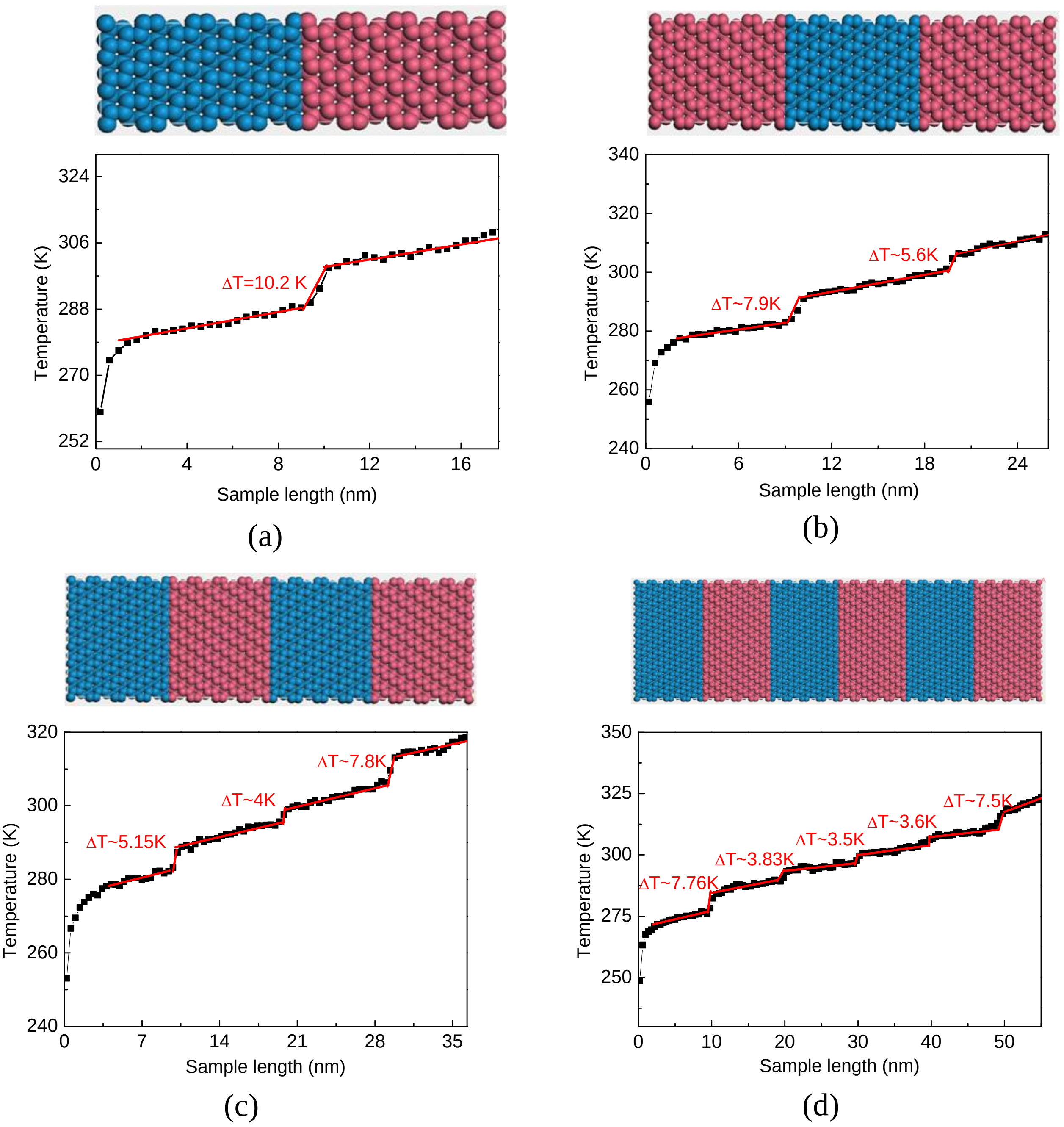

Figure S2. Variation of temperature profiles for twinned diamond (*D=9.92 nm*) with the variation of model sizes. (a) *L=19.84 nm*, (b) *L=29.76 nm*, (c) *L=39.68 nm*, and (d) *L=59.52 nm*.

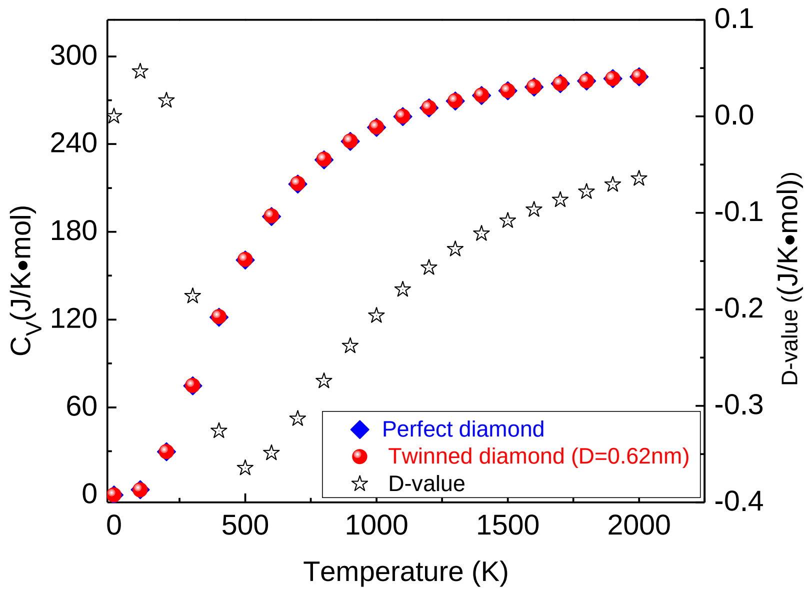

Figure S3. Calculated heat capacities for the twinned (*D=0.62nm*) and perfect diamonds at different temperatures, as well as D-values between perfect diamond heat capacities and twinned diamond heat capacities.

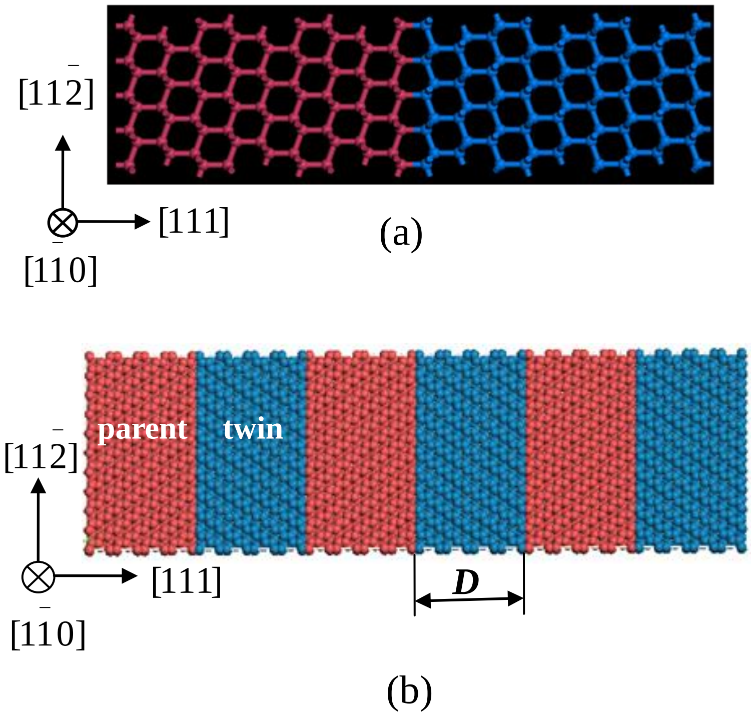

Figure S4. (a) Atomic arrangements of a $\sum 3(111)$ twin boundary. (b) Schematic representation for twinned diamond in simulation.

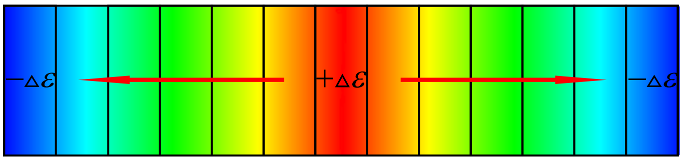

(a)

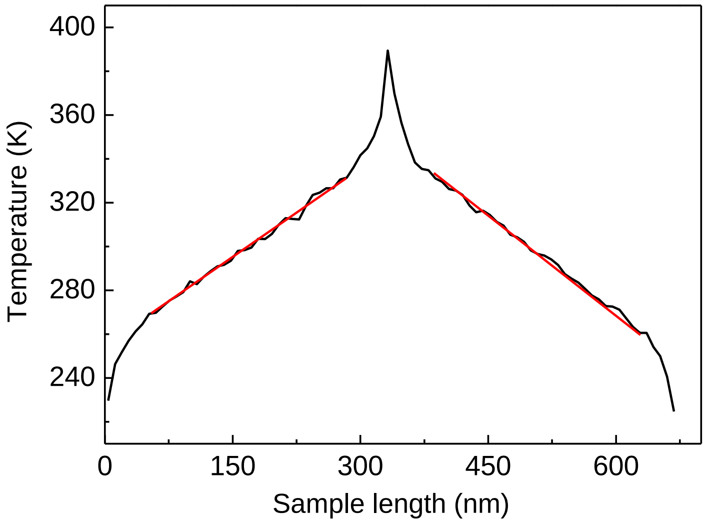

(b)

Figure S5. Schematic view of the calculation of thermal conductivity by NEMD method. (a) Diagrammatic sketch of the periodic simulation box as well as the locations of hot and cold regions for NEMD simulation of thermal conduction. (b) Obtained temperature profile along the heat transmission direction.
